# Necrotizing Enterocolitis: Overview on In Vitro Models

**DOI:** 10.3390/ijms22136761

**Published:** 2021-06-23

**Authors:** Luigia De Fazio, Isadora Beghetti, Salvatore Nicola Bertuccio, Concetta Marsico, Silvia Martini, Riccardo Masetti, Andrea Pession, Luigi Corvaglia, Arianna Aceti

**Affiliations:** 1 Department of Medical and Surgical Sciences, University of Bologna, 40138 Bologna, Italy; luigia.defazio2@unibo.it (L.D.F.); salvatore.bertuccio2@unibo.it (S.N.B.); concetta.marsico2@unibo.it (C.M.); silvia.martini9@unibo.it (S.M.); riccardo.masetti5@unibo.it (R.M.); andrea.pession@unibo.it (A.P.); luigi.corvaglia@unibo.it (L.C.); arianna.aceti2@unibo.it (A.A.); 2 Pediatric Oncology and Hematology “Lalla Seragnoli”, Pediatric Unit-IRCCS Azienda Ospedaliero-Universitaria, 40138 Bologna, Italy; 3 Neonatal Intensive Care Unit-IRCCS Azienda Ospedaliero-Universitaria, 40138 Bologna, Italy

**Keywords:** necrotizing enterocolitis, preterm infants, in vitro models, cellular lines, intestinal organoids

## Abstract

Necrotizing enterocolitis (NEC) is a gut inflammatory disorder which constitutes one of the leading causes of morbidity and mortality for preterm infants. The pathophysiology of NEC is yet to be fully understood; several observational studies have led to the identification of multiple factors involved in the pathophysiology of the disease, including gut immaturity and dysbiosis of the intestinal microbiome. Given the complex interactions between microbiota, enterocytes, and immune cells, and the limited access to fetal human tissues for experimental studies, animal models have long been essential to describe NEC mechanisms. However, at present there is no animal model perfectly mimicking human NEC; furthermore, the disease mechanisms appear too complex to be studied in single-cell cultures. Thus, researchers have developed new approaches in which intestinal epithelial cells are exposed to a combination of environmental and microbial factors which can potentially trigger NEC. In addition, organoids have gained increasing attention as promising models for studying NEC development. Currently, several in vitro models have been proposed and have contributed to describe the disease in deeper detail. In this paper, we will provide an updated review of available in vitro models of NEC and an overview of current knowledge regarding its molecular underpinnings.

## 1. Introduction

Necrotizing enterocolitis (NEC) is the most serious gastrointestinal (GI) disease of prematurity and the most common cause of death in extremely preterm infants aged 2 to 8 weeks of age [1]. NEC primarily affects infants born before 32 weeks of gestation and with low birth weight (BW); the lower the gestational age, the higher is the incidence of the disease [2]. Recent meta-analyses estimated that NEC occurs in 7% of low-BW infants in neonatal intensive care units. Mortality varies from 10 to 30% and, despite several decades of basic and clinical research, has remained largely unchanged [3]. NEC is characterized by inflammation and necrosis of the distal small bowel and proximal colon, with extensive infiltration of neutrophils leading to perforation, peritonitis, systemic sepsis, and multiorgan failure [4]. The main factors involved in the pathogenesis include functional and anatomical immaturity of both the intestinal barrier and the GI immune system, and abnormal patterns of intestinal microbial colonization [5,6]. A proper colonization of the gut is essential for intestinal barrier function and immune maturation [7]. The gut microbiota of preterm infants, together with a unique set of environmental exposures, is made of a dramatically lower number of beneficial species, lower bacterial diversity, and higher proportion of pathogens compared to healthy breastfed term infants [8,9]. Such dysbiosis may result in a hyperinflammatory response by the premature immune system, causing a violent inflammatory storm that leads to increased intestinal permeability, bacterial translocation, and inflammation [10]. Metabolites and products, such LPS, secreted from colonizing bacteria interact with Toll-like receptors (TLRs) on the intestinal epithelial cells (IECs) and on the submucosal inflammatory T cells. Once activated, TLR-4 in the IECs elicits the activation of several signal transduction pathways, including the inhibitor of NF-κB (IκB) kinase (IKK) [11], a critical upstream kinase that activates NF-κB. This leads to the production of many inflammatory mediators, including cytokines and chemokines, which induce immune cell recruitment, especially neutrophils, and intestinal inflammation. Specifically, the exaggerated expression of TLR4 in the intestinal epithelium of the premature infant leads to the induction of the lymphocyte chemoattractant CCL25 on the intestinal epithelium; in addition, the over-expression of TLR4 is associated with increased expression of pSTAT3 [12]. Recent studies have highlighted the role of immune cells in NEC pathogenesis, describing an imbalance between Tregs and Th17 cells with a consequent exaggerated pro-inflammatory state [12]. The elevation in pSTAT3 leads to the induction of a Th17 phenotypic response and reduction in Foxp3+ Treg cells, resulting in the polarization of T cells toward a Th17 phenotype.

I-17 released by CD4^+^ Th17 cells results in intestinal mucosal injury, as demonstrated by impaired enterocyte tight junctions (TJs), increased enterocyte apoptosis, and decreased enterocyte proliferation, all of which are hallmark features of NEC [13].

Human milk (HM) feeding is well known to constitute a protective factor against NEC in preterm infant [14], even if the exact mechanisms through which this effect is exerted are still to be described in detail. The bioactive components of HM, including soluble immune factors, antimicrobial proteins and peptides, functional fatty acids, hormones, oligosaccharides, and stem cells, are thought to act synergistically by exerting immunomodulatory, anti-infective, antioxidant, growth-promoting, and gut-colonizing effects. Several studies have documented substantial differences in the composition of gut microbiota between preterm infants fed HM and those fed formula milk, with HM leading to the highest microbial diversity as well as a more gradual acquisition of diversity compared to formula milk [15,16].

Although numerous animal models for NEC exist and have contributed to shed light on its complex pathophysiology, response to injury and therapeutic interventions may be highly variable across species. Furthermore, it is ethically challenging to study disease pathophysiology or novel therapeutic agents directly in human subjects, especially in vulnerable populations such as preterm infants. Thus, it would be highly desirable to develop novel in vitro models of NEC using human tissue, leading to a better understanding of complex intestinal pathophysiology and, therefore, to promising prevention or therapeutic strategies.

Hence, this narrative review is aimed at summarizing available in vitro studies about NEC pathogenesis and innovative approaches used to disentangle NEC molecular underpinnings.

## 2. Modeling NEC in IECs

IECs are widely used to study the effects of NEC-associated factors at the cellular level. Several different cell lines have been used as intestinal models to study NEC in experimental conditions. The human epithelial cell line Caco-2 constitutes the most widely used intestinal model for NEC. This cell line undergoes a gradual villus-like enterocytic differentiation process, spontaneously starting after 30 days of culture, which is like that observed in the epithelium of the intact fetal small and large bowel. The Caco-2 cell line shows common features of the small intestine. This cell line cannot differentiate into goblet cells; thus, one important limitation of such a model is the inability to produce mucus. The HT-29 cell line is derived from a colon adenocarcinoma and is considered a pluripotent intestinal cell line, as changes in the culture media can lead to different enterocytic differentiation paths. Unlike in the Caco-2 cell line [17,18], differentiation in HT-29 [19,20] is not spontaneous, but rather depends on nutritional and culture conditions [21]. When treated with sodium butyrate, HT-29 cells can differentiate into goblet-like cells, thus having the ability to produce mucus. IEC-6 and IEC-18 [22] cells are non-transformed intestinal cells derived from the rat small intestine and from native rat ileal crypts, respectively. Both these cell lines are characterized by an epithelioid morphology; due to the lack of staining for the villous enterocyte marker, these cells are described as having an immature crypt-like phenotype [23]. FHs 74-Int and H4 cells are the most frequently used model of human epithelium in NEC experimental studies. The H4 cell line [24] was developed from fetal small intestinal epithelial cells at 20–22 weeks gestation, whereas the FHs-74-Int [25] is derived from a 12–16-weeks-old human fetus.

The best characterized in vitro experimental NEC model is obtained by treating IEC lines with LPS, which was demonstrated to induce a dose-dependent increase in the expression of TLR4 in IEC-6 cells [26]. In other experimental settings, H_2_O_2_ was used to induce IECs injury, decreased cell viability, upregulated oxidative damage, and increased inflammation, thus mimicking the damage to the gut epithelium which is seen in vivo [27]. H_2_O_2_ treatment in IECs is a well-established model of epithelial injury induced by oxidative stress and it relies on the evidence that radical oxygen species (ROS) cause epithelial permeability changes and mucosal injury in small intestinal cell lines [28]. ROS-mediated IEC apoptosis is known to play a significant role in the pathogenesis of NEC in premature infants [29].

TNF-α and IFN-γ are the main cytokines associated with intestinal inflammation [30]. Since TNF-α and IFN-γ are key regulators of inflammation in experimental NEC, those cytokines were used in vitro in order to mimic the inflammatory conditions of this disease [30]. Another model used to mimic the features of NEC in vitro is *Cronobacter sakazakii* (CS). A Gram-negative, rod-shaped, and non-spore-forming pathogen in the *Enterobacteriaceae* family, it is commonly found in dairy products, and contamination of infant formula with CS was described in association with NEC outbreaks [31]. A role for CS in the pathogenesis of NEC has been postulated, as its introduction into experimental NEC models was shown to exacerbate intestinal injuries [32,33].

### 2.1. Overview of In Vitro NEC Studies

#### 2.1.1. Studies on Gut Permeability

A functional barrier including the mucus layer and TJ complexes (TJCs) limits the direct contact between pathogens and the epithelium while regulating the permeability of nutrients, water, and ions [34]. TJCs are specialized, multipurpose adhesion proteins that create a seal between neighboring IECs, whereas the mucus layer is the first line of defense from external molecules reaching the gut lumen. A disruption of the intestinal barrier is documented in NEC and several studies suggest that LPS, or bacteria which express LPS in their cell wall, increase the permeability across the intestinal epithelial layer by destroying TJCs. 

In this respect, several studies have investigated the role of various proteins which are involved in the function of the TJC, such as claudins, zonulin, and occludins. Claudins have been proposed as potential biomarkers for intestinal TJ integrity. There are different published studies examining the role of claudins in NEC and all the studies highlight a change in claudin expression following NEC and correlating with increased intestinal permeability [35,36]. Ares et al. [37] suggested that the increased permeability observed in LPS-treated Caco-2 cells occurs because of the upregulation of claudin-2. The claudin 2 overexpression in experimental NEC cells is accompanied by a change in the protein localization within the subcellular compartment, with a decreased expression of membrane-bound claudin 2 and an increased expression in the cytoskeleton compartment.

Zonulin has been proposed to modulate intestinal permeability by disassembling the TJCs in the intestinal epithelium [38]. Ling et al. [39] used Caco-2 cells exposed to LPS to study the role of zonulin in NEC-associated gut barrier dysfunction. Electron and immunofluorescence microscopy of LPS-treated Caco-2 cells showed a greater length of the apical and basolateral TJP and their different localization and fainter structure characterized by a decrease in zonulin, occludin, and claudin-3 proteins compared to control cells. 

Occludin plays an essential role in paracellular transport and regulation of macromolecules. Through its phosphorylation and dephosphorylation of serine/threonine residues, occludin changes the permeability of the cellular barrier [40]: in IECs, decreased occludin content is associated with increased barrier permeability [41]. Grothaus et al. [42] documented the importance of occludin in the maintenance of intestinal barrier function and integrity, showing that occludin gene expression is downregulated in human NEC samples. They observed that Caco-2 cell line exposed to LPS showed a decreased occludin expression, compared to controls. In addition, Lu et al. [43] observed that DRG1, a protein closely correlated with cell junctions, was constitutively expressed during the intestinal maturation process but significantly decreased in the ileum in the context of NEC. Data obtained from Caco-2 and FHs74int cell lines revealed that DRG1 deficiency destabilized the E-cadherin and occludin proteins near the cell membrane and increased the permeability of the epithelial cell monolayer. Fan et al. [31] observed that infection with *Cronobacter sakazakii* in HT-29 and Caco-2 cells reduced the transepithelial/transendothelial electrical resistance (TEER) of the monolayers in a time-dependent manner. *Cronobacter sakazakii* induced increased intestinal epithelial permeability in vitro, leading to the disruption of tight junctions by decreasing zonulin and occludin. In addition, the infection with *Cronobacter sakazakii* led to a downregulation of mucin production. 

The role of the Hippo–YAP (Yes-associated protein) signaling pathway in reparative response after tissue injury and junctional integrity has also been investigated. Goswami et al. showed that RAB11A, a small GTPase that mediates the anterograde cellular trafficking, controls the biochemical associations of YAP with multiple components of adherens and TJ, including α-catenin and β-catenin. After chemical injury to the intestine, mice deficient in RAB11A were found to have reduced epithelial integrity, decreased YAP localization to adherens and TJ, and increased nuclear YAP accumulation in the colon epithelium [44].

Recently, Chen at al. [45] investigated the role of phenazine biosynthesis-like domain-containing protein (PBLD) in intestinal barrier function defects and dysregulation of the intestinal immune response. The authors studied colonic tissue samples from patients with ulcerative colitis (UC) and constructed specific intestinal epithelial PBLD-deficient (PBLD^IEC−/−^) mice. PBLD was decreased in patients with UC and was correlated with levels of TJ and inflammatory proteins. PBLD^IEC−/−^ mice were more susceptible to chemical-induced colitis compared with wild-type (WT) mice. In the induced colitis model, PBLD^IEC−/−^ mice had impaired intestinal barrier function, greater immune cell infiltration in colonic tissue, and less TJ proteins than WT mice. Furthermore, NF-κB activation was markedly elevated and resulted in higher expression levels of downstream effectors (IL-6, TNF-α) in colonic epithelial cells from PBLD^IEC−/−^ mice than WT mice with colitis. Interestingly, PBLD overexpression in IECs consistently inhibited TNF-α/interferon-γ-induced intestinal barrier disruption and TNF-α-induced inflammatory responses via the interaction with IKKs [45].

Despite promising results from in vitro models of NEC examining gut permeability, which have implemented the understanding of the molecular mechanisms correlated with NEC pathophysiology, existing data are still insufficient to support a diagnostic or prognostic role of these biomarkers in NEC [46].

#### 2.1.2. Studies on Gut Inflammation

The gut of preterm infants with NEC is characterized by an upregulation of inflammatory mediators, including cytokines, which induces intestinal injury and necrosis. Specifically, LPS treatment of IECs induces an inflammatory response by increasing the expression of IL-6, IL-8, and TNF-α. Ling et al. [39] reported, after stimulation with LPS, an increase in IL-6 and TNF-α in Caco-2 cells and in TNF-α mRNA expression in IEC-6 cells, compared with controls [47]. In a H_2_O_2_-induced NEC model, the increase in IL-6 was observed in IEC-18 and Caco-2 cells [48]. It was observed that SOCS3, a key regulator of cytokine signal transduction, was dramatically decreased in NEC tissue samples [49] and that the deletion of SOCS3 led to rapid inflammation in mice [50]. To determine the effects of SOCS3 on inflammatory factors, Zhang et al. [51] detected the expression of TNF-α, IL-10, and IL-6 in enterocytes (H4 and Caco-2 cells) undergoing LPS stimulation. They observed that LPS inhibited SOCS3 expression and that overexpression of SOCS3 decreased the levels of TNF-α and IL-6, and increased the levels of IL-10. Taken together, these data underscore the impact of SOCS3 in the protection from LPS-induced inflammation in enterocytes. As an important late inflammatory mediator, high-mobility group box 1 (HMGB1) is closely related to the occurrence, development, and complications of NEC. HMGB1 protein is upregulated in the progression of NEC, and the inhibition of HMGB1 expression has been demonstrated to inhibit the TLR4/NF-κB signaling pathway, alleviating intestinal inflammation in rat and human IEC models of NEC. Interestingly, HMGB1 gene single-nucleotide polymorphisms (SNPs) were recently associated with the susceptibility of NEC and with the survival prognosis of newborns with NEC [52]. Furthermore, intestinal injury may initiate in utero, induced by maternal inflammation. In a rat model, LPS-induced maternal inflammation was shown to induce intestinal injury in offspring, with intestinal mucosal barrier disruption, and changes in the composition of the intestinal microbiota similar to the microbiota alteration found in human infants who develop NEC.

Conversely, the exposure of human fetal IECs to human milk has been proposed as having an anti-inflammatory effect [53]. An interesting study compared the inflammatory profile of human fetal primary IEC line H4 with those obtained from adult cell lines HT29-cl19A and Caco-2 treated with TNF-α or IL-1β. The study highlighted that the immature intestine had an elevated baseline inflammatory state compared to adult enterocytes and differences in the pattern of alteration of IL-8 secretion by human milk factors such as transforming growth factor (TGF-β1 and TGF-β2), erythropoietin, IL-10, and epidermal growth factor (EGF). TGF-β1 and Epo decreased both IL-8 secretion stimulated by TNF-α or IL-1β in human fetal IECs [54]. Human milk has been shown to be protective and to modulate cytokine expression in IECs, but the underlying molecular mechanisms have not been elucidated yet.

#### 2.1.3. Studies on Gut Cells Proliferation

Cytokines, such as IFN-γ and TNF, are known to induce sudden negative changes in the expression of the TJ proteins, but they can also induce cell death signaling. Autophagy, apoptosis, and necrosis are induced by multiple stress pathways, which may interfere with the intestinal barrier function, and they have been implicated in the pathogenesis of several GI diseases including NEC [55]. The effect of LPS on the proliferation of IECs was observed in different LPS-stimulated IECs. Zhang et al. [26] showed the inhibition of Caco-2 cells’ viability, suggesting that LPS inhibits cell growth and replication. Yuan et al. [47] observed an increased expression of the autophagy markers and a significantly higher TNF-α mRNA expression in IEC-6/NEC. The study suggests that TNF-α induced autophagy, thereby suppressing proliferation and promoting apoptosis in IEC-6/NEC cells. LPS is a physiological inducer of TNFα in various cell systems and its activation results in apoptosis and induction of inflammatory responses. The importance of TNF-α in the initiation and propagation of NEC has been well documented. Cyclic adenosine monophosphate (cAMP) and protein kinase A (PKA) are important mediators and regulators of apoptosis. Blackwood et al. [56] observed in IEC-6 and FHs 74 Int cells that both LPS and *Cronobacter sakazakii* caused an increase in cAMP and activation of PKA that contributed to cellular apoptosis. Apoptosis was even higher in FHs 74 Int cells than in IEC-6 cells, suggesting a higher susceptibility to inflammatory stimuli than adult IECs.

## 3. Intestinal Organoid Models

**Figure 1:**
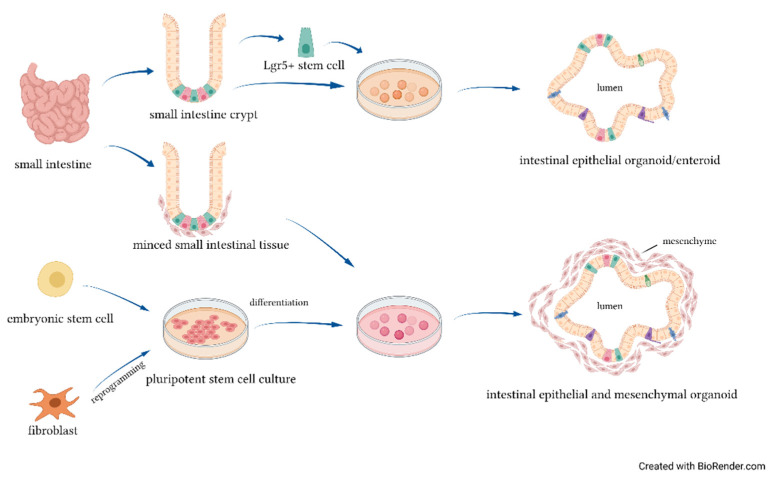
Intestinal organoids’ cellular sources.

An organoid (Figure 1) is a self-organizing tridimensional structure which exhibits similar organ functionality and physiological features as the tissue of origin [57]. Intestinal organoids mimic in vivo epithelial regenerative capacity, with apoptotic cells being continually released into the lumen of the organoid, while new cells are differentiated from the Leucine-rich repeat-containing G-protein-coupled (Lgr5+) cells within the crypts to restore the epithelium. Intestinal organoids can be derived from two sources of stem cells: organ-restricted adult stem cells (ASCs) and pluripotent stem cells (PSCs), both in the form of induced (iPSCs) and embryonic stem cells (ESCs) [58,59,60]. ASC-derived organoids are generated by harvesting stem cells containing crypts or isolating single Lgr5+ expressed cells from human or mouse gut tissue [61]. ASC-derived organoids contain only epithelial cell types derived from the crypt-based stem cells and are referred to as enteroids or colonoids, depending on whether they originate from the small intestine or the colon [62]. Lgr5+ stem cells can spontaneously proliferate and differentiate in the absence of any stromal and mesenchymal cells. The formation of villi, a major characteristic of the differentiated intestine, has not been achieved with enteroids but only with organoids [63]. These data suggest that the presence of mesenchyme may be needed to activate the genes involved in villus formation. Organoids can be derived from human intestine harvested at the time of resection for NEC or from primary intestinal tissue of animals subjected to experimental NEC, by isolating the crypts of terminal ileum. Stress factors, such as hypoxia and LPS, were used to induce intestinal epithelial injury in an organoids model. The intestinal organoids treated with NEC-associated stress factors showed increased inflammation similar to intestinal injury found in mice with NEC [39]. 

### Overview on Studies Using NEC-Organoid Models

Ares et al. [64] described the use of enteroids derived from human intestinal tissue samples as a novel ex vivo model for the study of NEC. The exposure of human intestinal enteroids to LPS caused an inflammatory response leading to histologic, genetic, and protein expression alterations similar to those found in human NEC. The LPS-treated enteroids experienced more apoptosis and an increased expression of Toll-like receptor 4 (TLR4) than controls. Another study on the NEC-enteroid models was conducted by Kovler et al. [65]: the authors harvested enteroids from a mouse intestine and simulated NEC features through the enteroids’ exposure to hypoxia and pathogenic bacteria. In NEC enteroids, the differentiated intestinal epithelium showed a disruption in tissue architecture, as documented by E-cadherin localization, an increase in inflammatory cytokines, and an upregulated activation of necroptosis. Similarly to what has been observed in the murine models [66], the single exposure of enteroids either to hypoxia or NEC bacteria did not induce such alterations, indicating that both components are probably necessary for a precision-based ex vivo NEC model. Lanik et al. [67] reported a decreased proliferation in enteroids derived from neonatal mice and premature infants in the presence of LPS. Importantly, the study suggested that breast milk restored the enteroid proliferation even in the presence of LPS, as demonstrated by the overexpression of Ki67, a protein strictly associated with cell proliferation. In a study conducted by Senger et al. [68], human fetal-derived (FEnS) and adult-derived enterospheres (AEnS) were generated from mice with NEC. Compared to controls, NEC-derived organoids were smaller, indicating less proliferation, and had more budding, suggesting higher differentiation. In addition, organoids cultured without Wnt medium showed a reduced proliferation and increased differentiation compared to the organoids cultured in medium with added Wnt. A similar growth response was observed in organoids derived from human NEC tissue, indicating that Wnt deficiency can lead to NEC-like injury in organoids. Several studies reported [63,68,69] that the Wnt and β-catenin pathways were dysregulated in organoids, in experimental NEC, and most importantly in human NEC. This deregulation leads to the impairment of intestinal epithelial stem cell proliferation and differentiation and leads to reduced enterocyte proliferation in response to TLR4 activation, as shown in organoids from TLR4-deficient mice [70]. NEC organoids also showed a lower expression of proliferation marker *PCNA* and ISC, genes associated with the innate immune response, intestinal epithelium maturation, and gut barrier function. Genes associated with Paneth cell antimicrobial activity, stem cell function, and the innate immune response (including inflammasome-related chemokines and cytokines, cytokine receptors, and TNF-related and CXCL8/IL-8 genes) were upregulated in a subgroup of FEnS. Although, the fetal-specific claudin-6, JAM3, claudin-2, and claudin-15 were highly expressed in all the FEnS but not in the AEnS. Li et al. [63] incubated organoids from fresh terminal ileum of newborn mice pups in a hypoxic condition and administered LPS to mimic NEC-like neonatal intestinal injury. Hypoxia and LPS exposure induced intestinal inflammation, as indicated by increased gene expression of pro-inflammation cytokines IL-6 and TNF-α Moreover, the immunostaining pattern of tight junction marker ZO-1 and claudin-3 showed the relocation of tight junction marker from the luminal to the outside membrane of the organoids. 

Intestinal organoids can be derived from two sources of stem cells: ASCs and PSCs, both in the form of iPSCs and ESCs. ASC-derived organoids are generated by isolated Lgr5+ stem cells or whole intestinal crypts and contain only epithelial cell types derived from the crypt-based stem cells. Intestinal epithelial and mesenchymal organoids can be derived from minced pieces of intestinal tissue or from PSCs.

## 4. Human Gut-on-a-Chip

Organ-on-a-chip (OoC) is a microengineered platform that aims to develop an enhanced in vitro model by mimicking the complexity of native tissues [71], overcoming many of the limitations of previous in vitro models. Chips incorporate microfluidic channels lined by living human cells and utilize a microfluidic approach to supply tissues with the nutrients and factors needed for their functions. Through a combination of cell biology, engineering, and biomaterial technology, the microenvironment of the chip simulates tissue interfaces and mimics the complex physical and biochemical microenvironment of living human organs [72,73]. In recent years, OoC has been proposed as a novel cell-based assay tool in the μTAS (Micro Total Analysis Systems) research field and has emerged for its ability to fill the large gap between in vivo and in vitro conditions. OoC responds to the need for modeling and testing platforms that would be more predictive of human responses [74]. These microdevices can be used to test and to create in vitro models of human disease, to closely monitor cell-level and tissue-level events [75], simulate and reverse pathological situations, and study the effect of etiological factors on tissue [76]. In order to further understand gut physiology, the intestinal microbiota, and disease processes, a novel technology primarily based on microfluidics and cell biology, called “gut-on-chip” (GOC), was developed to simulate the structure, function, and microenvironment of the human gut [77]. It is well established that NEC is a complex and multi-factorial disease [78]. Gut-on-chip platforms could be employed to simplify the complexity of human intestine and could provide a robust modular platform for NEC studies, for analysis of intestine–microbiota interactions, etiology, and development of intestinal inflammation, but also for defining potential therapeutic targets and testing drug candidates for NEC treatment.

### Overview on Studies Using Human Gut-on-a-Chip and Future Prospects for NEC

GOC models, based on established human intestinal epithelial cell lines (Caco-2 [17,18] or HT-29 [19,20] cells) growing on extracellular (ECM)-coated surfaces, have been used to study barrier function, intestinal cell and tissue morphology, or intestinal differentiated functions. In several studies, chip-based models were used to study gut inflammation. Kim et al. showed that LPS endotoxin elicited an increased secretion of proinflammatory cytokines such as interleukin IL-1β, IL6, IL-8, and TNF-α. That inflammatory storm is necessary and sufficient to compromise intestinal barrier function, as demonstrated by increased expression of intercellular adhesion molecule (ICAM)-1 and villous blunting [73]. Shin et al. used a gut chip-based model inspired by dextran sodium sulfate (DSS)-induced colitis models in mice in order to identify the initiating factors in gut inflammation. They demonstrated that epithelial barrier dysfunction, pathogenic bacteria (LPS), and immune cells (PBMC) were required to achieve gut inflammation [79]. GOC have been engineered with increasing complexity that also includes neighboring channels lined by immune cells, commensal microbes, pathogenic bacteria, and human microvascular endothelium. Gut microbiota study has been limited to genomic and metagenomic analysis given the difficulty in culturing the largely anaerobic bacterial population. In a recent study, a human GOC microdevice was used to coculture multiple commensal microbes in contact with living human intestinal epithelial cells and to analyze how gut microbiome and inflammatory cells contribute to intestinal bacterial overgrowth and inflammation [80]. Incorporation of the gut microbiota on-a-chip could help answer many questions previously unanswerable, such as how microbial composition differs on the cell surface versus lumen, how specific bacteria interact with host cells, and tracking microbiota changing over time in response to different stimuli. The presence of immune cells in the GOC model provides an efficient approach to study the interaction between intestinal and immune cells. Shah et al. [81] were able to co-culture human epithelial cells and microbial cells with primary human CD4^+^ T cells in a single microfluidic device. The device was used to examine the gut response to the subsequent interactions with the immune system. The lack of the cellular immune component, which plays a key role in the multifactorial landscape of NEC pathogenesis [12], could be a limit of the chip system because of the impossibility to study the interaction between immune and epithelial cells and the role of immune circulating factors. The presence of the endothelium cells in the GOC model allows analysis of the migration of immune cells. Since migration is regulated by the vasculature [82], interactions between immune cells and endothelial cells were studied by Han et al. [83], who used an inflammation GOC model to show the transendothelial migration of neutrophils. Furthermore, the relevance of angiogenesis to epithelial development/function and the importance to incorporate this into a novel GOC system was demonstrates by Sailer et al. [84].

## 5. Conclusions

Despite continuous progress in neonatal intensive care, NEC remains a devastating disease for premature infants, with few specific treatment options. Clinical and scientific research studies have identified risk factors for NEC and experimental animal models have obtained significant clues on the biological foundations of the disease. Despite continuous scientific advancements, the pathophysiological underpinnings of this disease remain incompletely understood. In vitro models of NEC have provided important tools to observe the molecular mechanisms involved in the disease, such as cell proliferation, apoptosis, and gene expression variation, and can serve as low-cost screening platforms for the discovery of the effect of new therapies and protective strategies, including human milk, and their mechanisms. Overall, existing experimental data suggest that inflammatory response, cell apoptosis, oxidative stress, gut permeability, and signal transduction differ significantly between NEC models and controls. In vitro models have the advantage of overcoming the difficulty in identifying cellular and molecular contributors to disease, but disadvantages include the lack of an immune component, which appears to be fundamental for the development of NEC [12]. So far, there are only a few published studies modeling NEC reproducing the characteristics of intestinal epithelium of the preterm infant. A comprehensive model of this disease is yet to be generated, despite continuous advancements toward the development of stress factors that mimic those seen in human NEC. Organoids overcome many of the limitations of standard cell lines, as they can more closely reflect normal physiology or disease pathogenesis and, to date, organoids from human intestine are one of the most promising tools for the development of a complete in vitro NEC model. Moreover, the development of the gut-on-a-chip model, incorporating not only GI epithelium but also immune, vascular, enteric, nervous, and other components of the GI microenvironment, could provide new insight into NEC disease mechanisms.

## Data Availability

Not applicable.
